# Effect of Phenotype Selection on Genome Size Variation in Two Species of Diptera

**DOI:** 10.3390/genes11020218

**Published:** 2020-02-19

**Authors:** Carl E. Hjelmen, Jonathan J. Parrott, Satyam P. Srivastav, Alexander S. McGuane, Lisa L. Ellis, Andrew D. Stewart, J. Spencer Johnston, Aaron M. Tarone

**Affiliations:** 1 Department of Entomology, Texas A&M University, College Station, TX 77843, USA; Jonathan.Parrott@asu.edu (J.J.P.); sps257@cornell.edu (S.P.S.); mcgu2014@tamu.edu (A.S.M.); lellis@hbu.edu (L.L.E.); spencerj@tamu.edu (J.S.J.); tamlucilia@tamu.edu (A.M.T.); 2 Department of Biology, Texas A&M University, College Station, TX 77843, USA; 3 School of Mathematical and Natural Sciences, Arizona State University, Glendale, AZ 85306, USA; 4 Department of Molecular Biology and Genetics, Cornell University, Ithaca, NY 14853, USA; 5 Harris County Institute of Forensic Sciences, 1861 Old Spanish Trail, Houston, TX 77054, USA; 6 Department of Biology, Houston Baptist University, Houston, TX 77074, USA; 7 Department of Biology, Canisius College, Buffalo, NY 14208, USA; stewar34@canisius.edu

**Keywords:** artificial selection, stabilizing selection, body size, development time, *Drosophila*, *Cochliomyia*, blow fly

## Abstract

Genome size varies widely across organisms yet has not been found to be related to organismal complexity in eukaryotes. While there is no evidence for a relationship with complexity, there is evidence to suggest that other phenotypic characteristics, such as nucleus size and cell-cycle time, are associated with genome size, body size, and development rate. However, what is unknown is how the selection for divergent phenotypic traits may indirectly affect genome size. *Drosophila melanogaster* were selected for small and large body size for up to 220 generations, while *Cochliomyia macellaria* were selected for 32 generations for fast and slow development. Size in *D. melanogaster* significantly changed in terms of both cell-count and genome size in isolines, but only the cell-count changed in lines which were maintained at larger effective population sizes. Larger genome sizes only occurred in a subset of *D. melanogaster* isolines originated from flies selected for their large body size. Selection for development time did not change average genome size yet decreased the within-population variation in genome size with increasing generations of selection. This decrease in variation and convergence on a similar mean genome size was not in correspondence with phenotypic variation and suggests stabilizing selection on genome size in laboratory conditions.

## 1. Introduction

Genome size is considered to be at the intersection of genotype and phenotype, as it has the full complement of DNA sequence, but is a physical measure of the amount of DNA, often recorded in mass (pg) [1,2]. Interspecific variation in genome size is commonly measured, which has revealed a 7000-fold variation in genome size between different animal species [3]. Less, however, is known about intraspecific genome size variation. Genome size is generally considered heritable and maintained for a species [2]. At a finer scale, though, that viewpoint is changing. With increased resolution in estimation techniques, there is now information on levels of intraspecific variation that show measurable differences in genome sizes between inbred strains [4,5], within populations [6], and between populations [7]. There, it is of great interest that the differences between these strains and populations are quantitative, can be visualized when co-prepared, and have been found to be related to fitness parameters [4,5,7,8].

While genome size is not related to organismal complexity, it has been found to have phenotypic and physiological correlates, which selection may act upon [1,9,10]. An increase in genome size can lead to an increase in overall cell size, through an increase in the physical volume of the nucleus and the cell in order to hold the larger quantity of genetic material [11,12]. This pattern has been found in both plants and animals [13,14,15,16]. While there are cellular correlations with genome size, the relationship may decrease with increases in scale of the phenotype [17]. For example, body size variation in *Drosophila* species is commonly related to cell-counts, but may be related to cell size [18] as a correlate of genome size. In the cases of flatworms and copepod crustaceans, where there is a significant relationship between genome size and body size, an increase in cell volume is accompanied by increase in cell number, suggesting that body size may be an interaction between cell size and count [19,20]. If this is the case, then genome size increases that affect cell size may be considered as one possible mechanism by which an organism can evolve a larger body size.

Increased genome size may also lead to increased cell-cycle time, replication times, and, in some cases, an increased development time [12,21,22,23,24,25,26]. There have also been multiple examples of where genome size is related to development rate in copepod crustaceans [27,28]. Additionally, genome size was found to be positively associated with body size in aphids, although their overall small genome size may be related to their rapid development times [29]. In passerine birds, genome size is not only positively correlated to cell/nucleus size, but is also positively related to wing loading index, defined as the amount of mass carried by unit wing area [13]. These associations suggest to some that genome size change may be in response to life history and ecological conditions [30,31,32]. It remains possible, however, that there is no direct, or general phenotypic consequences of variation in genome size, or that significant associations are mitigated through some other third factor [17]. The generality of a phenotypic correlation with genome size remains to be established. 

Given the relationships explained in the above paragraphs, we expect that if there is a phenotypic or fitness correlation between a selectable trait and genome size, then differential selection on that trait may result in an indirect selection for a change in genome size; either as an increase or a decrease in the amount of DNA. Alternatively, if genome size is not correlated or indirectly selected, any genome size change that occurs (if any), including direction (increase/decrease), should be independent of the change in the selected trait. Furthermore, the variation in genome size should not be tied to variation in the selected trait. Finally, genome size may be related to fitness, where that fitness is associated with body size or development rate, yet it is constrained by multiple other pleiotropic fitness effects related to genome size variation. In this last case, we might see evidence for stabilizing selection, and we may see genome size change only when the effects of antagonistic selection are limited by a small effective population size, as proposed by Lynch and Conery [33]. 

Here we use long-running selection experiments for development time in the blow fly *Cochliomyia macellaria* and a longer running selection experiment for differential body size in *Drosophila melanogaster* to investigate the effects of developmental and phenotypic selection on genome size. If there is a strong relationship between genome size and development time in *C. macellaria*, we expect to see a decrease in genome size with faster development times and an increase in genome size with longer development times. Similarly, if a strong relationship exists between genome size and body size, we expect to see larger genome sizes in *D. melanogaster* that are selected for large body size, and the opposite for those selected for small body size. Any other change in genome size will argue for either (1) genetic drift and neutral genome size evolution, or (2) constraints on genome size evolution imposed by antagonistic selection, due to multiple pleiotropic fitness effects.

## 2. Materials and Methods 

### 2.1. C. macellaria Colony Development

Wild type *Cochliomyia macellaria* were collected from three locations in Texas in order to replicate selection regimes: College Station, Longview, and San Marcos. Colonies were established at the Forensic Laboratory for Investigative Entomological Sciences (FLIES) at Texas A&M University. As part of a larger experiment, three selection treatments were established for each origin: slow development, fast development, and a selection control. Specimens from this selection experiment were vouchered at the Texas A&M University Insect Collection (Voucher #728).

*C. macellaria* eggs (*N* = ~1200) were collected for each location and reared in one-quart mason jars with sand and beef liver. Time from egg laying to adult emergence was measured in hours for each generation of selection. Rearing took place in a Percival incubator at 25 °C and 14:10 Light-Dark Cycle at 40% relative humidity. The first 200 of the 1200 flies in generation one were used to establish fast developing colonies. The last 200 flies to emerge were used to establish slow developing colonies. From this point, fast flies were selected as the first 200 to emerge, whereas slow flies were the last 200 to emerge. Control flies were reared the same as selected flies, but no selection on development time was imposed between generations. Selection of flies for fast, slow and controls were carried out each generation for 32 generations and development times were recorded each generation. This resulted in nine different populations: Slow, Fast, and Control development time for populations from three different cities. Adult flies from each generation were frozen at −20 °C for further genetic analyses, a subset of which were used for this study.

### 2.2. Drosophila Body Size Selection

Selection lines, or outbred populations, were derived from the same populations evaluated in Turner et al. [34] and subsequently in Stewart and Rice [35], where full details of selection protocols and the selection response can be found. For these purposes, we utilized three of the lines described in these papers, specifically the “Large” (both males and females experienced strong artificial selection for increased body size), “Small” (both males and females experienced strong artificial selection for decreased body size), and “Control” (no artificial selection on body size in either sex) lines. Within each treatment, two independent replicates lines (1 and 2) were maintained, resulting in six lines (L1, L2, S1, S2, C1, C2). For the purpose of this manuscript, flies were taken for genome size and cell-count measurement after 220 generations of selection, henceforth referred to as the outbred selection lines. Additionally, for this study, after 130 generations of size selection, inbred lines were formed from the size selected lines (L1, L2, S1, S2) through 10 generations of sibling mating. The control population (C1, C2) were included in the analysis of genome size of outbred selection lines but did not have subsequent isolines developed. A critical distinction between the *Drosophila* and the blow fly experiment is that the census size, and therefore the effective population size, was carefully maintained in the body size selection experiment—specifically each generation, was cultured with 320 breeding individuals at a 50:50 sex ratio. Previous studies have found this to only have modest effects on the effective population sizes of these flies [36]. In the case of the *C. macellaria* experiment, we expect a greater decrease of the effective population size each generation due to the process of artificial selection in a closed population and eventual inbreeding effects.

### 2.3. Species Genome Size Estimation and Calculation of Cell-Count Ratio

Individual *C. macellaria* or *D. melanogaster* were co-prepared with a standard female *Drosophila virilis* (1C/Haploid genome size = 328 Mbp) for flow cytometric analysis of genome size [37]. Briefly, neural tissue was dissected for both the sample and the standard, placed into 1 mL of Galbraith buffer within a 2 mL Kontes dounce tube and ground with 15 strokes of a Kontes B pestle. The sample was then filtered through 43 µm nylon mesh and stained for at least 20 min using 25 µL of 1 mg/mL concentrated propidium iodide solution. Genome size of the sample was estimated by comparing the position and mean of the fluorescent peak between the sample and the standard. Samples that were degraded when frozen, as evidenced by low nuclei counts, were discarded. In *C. macellaria*, genome size was measured in multiple individual frozen samples collected for each location (College Station, Longview, San Marcos) at three generational time points (1, 10, 32). Results from individual male and female *C. macellaria* values were pooled for post-estimation analyses, as there is not a statistical difference in genome size between the sexes and a small effect size of sex on development time, with only about five-hour difference in development time between males and females in control lines [38]. Only female *D. melanogaster* were used for estimation for the outbred S1, S2, C1, C2, L1, and L2 selection populations. Male and female genome sizes were estimated separately for the large and small isolines.

The cell-count ratio for *D. melanogaster* was estimated by taking the ratio of the number of counts in the *D. melanogaster* 2C (diploid) peak to the number of counts in the standard *D. virilis* 2C peak. The cell-counts were taken from the same flow cytometric peaks used for genome size estimation. Samples were taken from whole heads of both species; thus, the cell-count ratios reflect numbers of cells in *D. melanogaster* heads compared to the reference *D. virilis* strain heads.

### 2.4. Statistical Analysis

#### 2.4.1. *C. macellaria*

Genome size was compared for each location (College Station, Longview, San Marcos) among treatments (Fast, Slow, Control) at three generational time points (1, 10, 32) using ANOVA in R 3.3.0 [39]. Tukey’s HSD was utilized in cases where significant ANOVA values were found. Genome size and development time data was also investigated with a variance component analysis using the anovaVCA function in the package VCA in R to determine the amount of variation at each generation [40]. The variance component analyses were completed using the model: Trait = City + Selection + City*Selection + Error. 

#### 2.4.2. *D. melanogaster*

Genome size and cell-counts were compared for isolines from different selection treatments and for the larger selection populations utilizing ANOVA in R 3.3.0 [39]. Tukey’s HSD was calculated and plotted onto boxplots with the confidence level set to 0.95. A two-dimensional Kolmogorov-Smirnov test was performed to determine if significant differences existed between distributions of large and small isolines separated by cell-count ratio and genome size using the function peacock2() in the package ‘Peacock.test’ in R [41].

## 3. Results

### 3.1. C. macellaria after 32 Generations of Selection

**Figure 1:**
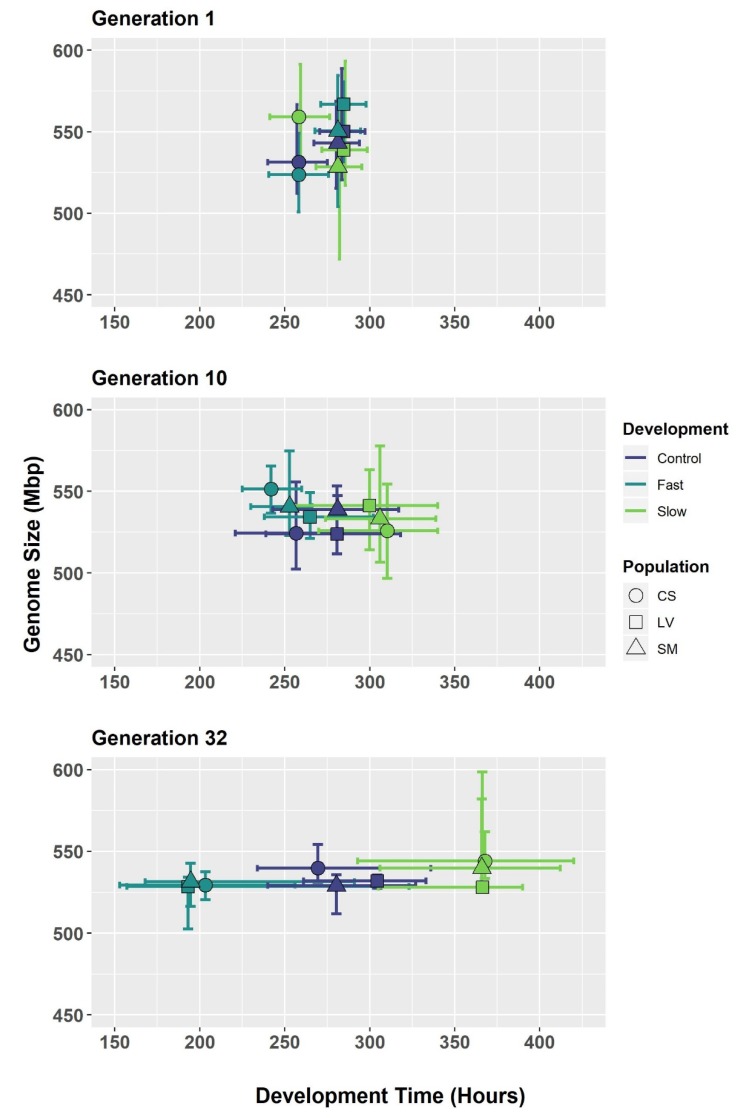
Range of development time and genome size for generations 1, 10, and 32 for different populations of the blow fly *Cochliomyia macellaria*. Hours of development time are plotted on the *X*-axis and genome size (Mbp) is plotted on the *Y*-axis. Points represent mean of the phenotype and lines represent the range of each trait. Colors represent development and shapes represent origin city. No change in development time by generation was seen in control lines, increases in development time was seen in slow selected lines, and decrease in development time was seen in fast selected lines. Variation in genome size reduced with generation and converged on a mean size of approximately 530 Mbp. Variation in development time increased from generation one and was maintained.

*C. macellaria* populations showed differential responses to selection, showing that populations selected for fast selection time resulted in faster development times and those selected for slower generation times developed slower (Figure 1 and Figure S1). According to the variance component analysis, an increase in variance for selection treatment was seen by generation for development time, verifying this divergence in phenotype (Table 1). Variance did not decrease in development time for the City*Selection interaction by generation (Table 1). Variance at generation 1 is zero due to development times being equal in starting populations (Table 1 and Table 2).

Average genome size measurements for each location, selection treatment, and generation is given in Table 2. After 32 generations of selection for fast and slow development, genome size change was observed in only four groups: College Station Slow, College Station Fast, Longview Control, and Longview Fast (Figure 1 and Figure S2, Table 2). In the College Station group, generation 10 differed significantly from the others (Tukey HSD, confidence level = 0.95, Figure 1 and Figure S2). Since Generation 1 and 32 were not significantly different in College Station groups, there was no directional change in genome size after selection. In the Longview groups, Generation 1 was different from the others (Tukey HSD, configuration level = 0.95, Figure 1 and Figure S2). Both control and fast groups changed in a downward trajectory (Figure 1). However, Generation 1 in Longview fast may be inflated due to a few individuals with larger genomes. Significant differences in genome size by generation were not found in any other group or at any other level.

While no directional change was seen in genome size after 32 generations of selection in any of the investigated strains, there was a decrease in the amount of variation for the City*Selection interaction with increasing generations (Table 1 and Table 2). This pattern of reduced variation in genome size in later generations is evident in the individual treatments (Figure 1) and when the genome sizes are pooled by generation (Figure S3). A reduction in variation of the selected trait, development time, in the City*Selection interaction by generation was not seen, however (Table 1).

### 3.2. Drosophila Genome Size after Size Selection

**Figure 2:**
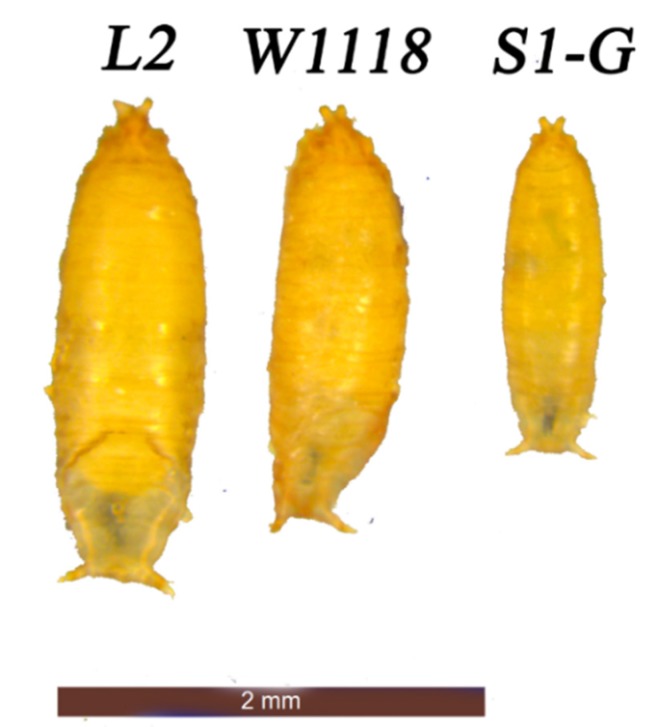
Pupal cases for *D. melanogaster* selected for large and small body size. All strains from this picture were maintained together, fed from the same batch of medium and the vials established for this image were started on the same day.

*D. melanogaster* selected for more than 130 generations for body size show dramatic differences in body size when compared to a control w1118 *D. melanogaster* stock, demonstrating success of selection (Figure 2). See Turner, Stewart, Fields, Rice and Tarone [34] and Stewart and Rice [35] for details of responses.

**Figure 3:**
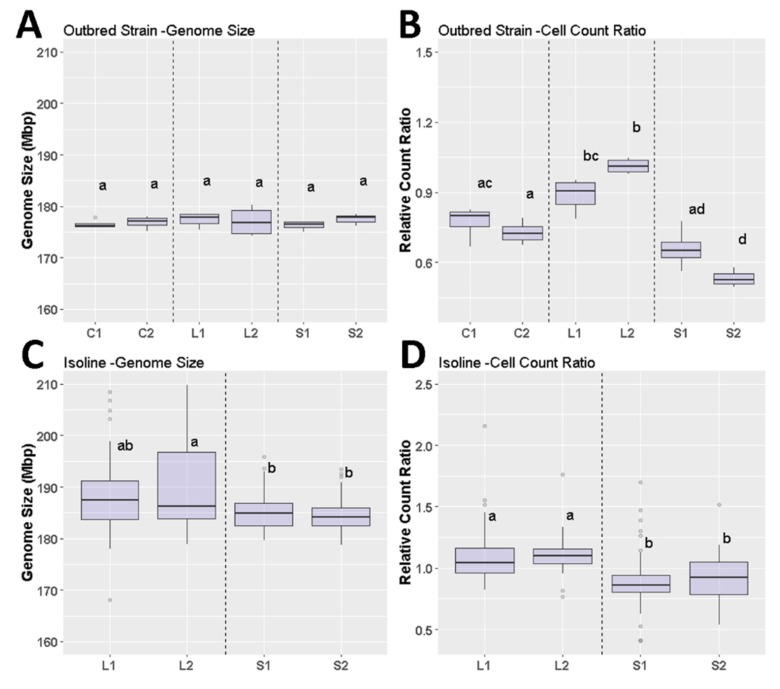
Boxplot comparisons of genome size and cell-counts between *D. melanogaster* differentially selected for body size. Genome size and cell-counts plotted by strain for outbred strains and isolines. Different letters above each box represent values significantly different according to Tukey HSD at the *p* < 0.05 level. (**A**) Genome size variation for outbred populations in control lines (C1, C2), large selected lines (L1, L2), and small selected lines (S1, S2). (**B**) Variation in cell-count ratio for outbred populations in control lines (C1, C2), large selected lines (L1, L2), and small selected lines (S1, S2). (**C**) Genome size variation for isolines developed from outbred populations for large-body size selected lines (L1, L2) and small-body size selected lines (S1, S2). (**D**) Variation in cell-count ratio for isolines developed from outbred populations for large-body size selected lines (L1, L2) and small-body size selected lines (S1, S2).

**Figure 4:**
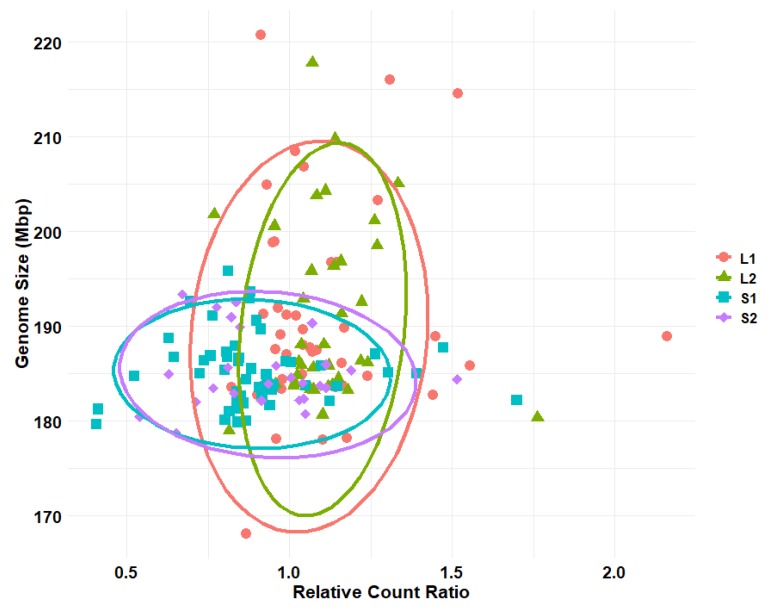
Distribution of genome sizes and relative cell-count ratio for large and small body size selected lines of *D. melanogaster*. Relative cell-count ratio (*X*-axis) plotted against genome size in Mbp (*Y*-axis) for large and small body size selected isolines. Large body size selected lines are represented in red circles (L1) and green triangles (L2), small body size lines represented in blue squares (S1) and purple diamonds (S2). Ellipses represent 95% confidence ellipses determined using the stat_ellipse() function in the ggplot2 package of R. Only a subset of large body size selected *D. melanogaster* isolines showed an increase in genome size. A two-dimensional Kolmogorov-Smirnov test using the function peacock2() in the package ‘Peacock.test’ found the distributions of large and small body size flies to be significantly different (*p* < 0.001) [41].

Selection for body size in *D. melanogaster* produces large and small individuals that differ significantly from unselected control lines [35] (Figure 2). Genome size estimates for each body size selected isoline of *D. melanogaster* and for the outbred population of each line is given in Table S1. When comparing genome sizes for males and females of each isoline, no significant differences were found with ANOVA and Tukey HSD. For lines maintained at larger population sizes, there was no statistical difference in genome size between *Drosophila* selected 220 generations for large or small body size according to an ANOVA (Figure 3). When comparing mean genome size among large and small body isolines, large body size selected *D. melanogaster* were found to have, on average, significantly larger genomes than those selected for small body size (ANOVA, *p* < 0.01, Table S1, Figure 3 and Figure 4). 

No significant differences were found in genome size among outbred populations of body size selected *D. melanogaster* lines (ANOVA, *p* = 0.897), yet there were significant differences in cell-count ratios (ANOVA, *p* < 0.0001). There were significant differences in genome size between isolines of large and small body size selected *D. melanogaster* (*p* < 0.01). There were, however, no significant differences between sexes of each isoline, so sex was not included in further analyses. In general, isolines experienced a bloating of genome size compared to outbred parental strains (~188 Mbp for females in the isolines compared to ~177 Mbp for females from the outbred lines). There were also highly significant differences in cell-count ratios between isolines selected for large and small body size (*p* < 0.0001). When the population is selected for large body size, there is an increase in the cell number (scored as cell-count ratio), whereas selection for a smaller body size decreases the cell-count. The distribution of genome sizes versus relative cell-count differences is shown in Figure 4, showing that a subset of large selected *D. melanogaster* also exhibited elevated genome sizes, while most exhibited increased cell-counts. A two-dimensional Kolmogorov-Smirnov test of these distributions found significant differences between large and small isolines when distributed by genome size and cell-count ratio (*p* > 0.001, Figure 4).

## 4. Discussion

Genome size has been found to be positively correlated to development time in some organisms, with increased development time with increased genome size [21,22,23]. However, to date, this relationship has not been explicitly demonstrated in higher Diptera. If there is a strong relationship between genome size and development time in Diptera, it would be expected that genome size would increase in flies selected for slower development and decrease in flies selected for faster development. After 32 generations of selection for fast and slow development in the blow fly *C. macellaria*, we find significant differences in development time as a phenotype. We find no change in mean genome size in any of the three populations selected for development time. It is hypothesized that species with slower development times have higher tolerances for increases in noncoding DNA [42]. Given this hypothesis, the selected slower development time may reduce the constraint on genome size, which may allow an increased genome size through time, but the resolution and the time of this study may not be high enough or long enough to detect this potential change.

While mean genome size was not found to be significantly impacted by selection for development time, the amount of variation in genome size decreased after selection regimes in each population of *C. macellaria*. A reduction in variation of a trait in a closed selection experiment of this size is not surprising—and is in fact expected [43]. The reduced effective population size at each selection event, each generation, acts as a bottleneck which reduces the genetic diversity and variation in each subsequent generation. This is demonstrated here with decreasing variance and error seen for genome size (Table 1). However, this pattern of losing variance was not seen when investigating the variance components for the development time phenotype of these same populations (Table 1). What is unknown, is how the amount of genetic variation in these populations has been maintained or decreased throughout time. In order to address this, individuals from each treatment and a generation of interest must be sequenced and compared, something which is currently out of the scope of this study.

Interestingly, with decreasing variation in genome size, the genome size of all selection treatments and populations converged on the same mean, approximately 530 Mbp (Figure 1, Figures S2 and S4). If the change in genome size was due to drift from low effective population sizes and bottleneck events, a reduction in genome size variation in each population would be expected and it would be expected that each population and treatment would result in random genome sizes from the original distribution of genome sizes. However, even though each of the three founding locations (College Station, Longview, and San Marcos) were subjected to three different selection treatments (Slow, Fast, and Control), and initially exhibited differences in genome sizes; all nine populations converged on the same genome size. This convergence on one genome size suggests that lab maintenance or inbreeding may be indirectly imposing stabilizing selection on genome size, resulting in a convergence on a non-extreme genome size, rather than random genome sizes as expected by processes such as genetic drift. The impact of lab maintenance or inbreeding may therefore obscure our ability to detect an effect of genome size change in this selection process.

Genome size is known to have effects at the cellular level, which affect cell size and volume. However, there is limited evidence that this change in cell size and volume scales to higher phenotypic levels. Increases in cell size have been found to be a factor in increased body size in some populations of *Drosophila subobscura*; however, differences in size are attributed to cell-count in other populations [18]. Here, selection for body size in *D. melanogaster* has resulted in significantly different body sizes and other phenotypic differences between these populations [34,35]. However, no significant differences in genome size were found between the replicated populations selected for large and small body sizes (Figure 3). However, there was a significant difference in relative cell-count ratio between these populations, with higher cell-counts in the large body size selected flies. Therefore, cell-count, and not genome size, is a significant contributor to body size change in this experiment with *D. melanogaster*. The relationship between body size and cell-count is common among many other organisms [44], and this suggests that changes in body size, while being heritable, is more easily dictated through variation in cell-counts than by large structural changes in the genome.

Genome size and cell-count ratios were also compared in isolines developed from the large and small body size selected *D. melanogaster*. As expected, and supportive of the above results, significant differences in cell-counts were found between large and small body isolines. In contrast, significant differences were found in genome sizes between these isolines, which all exhibited larger genome sizes than their parent strains (Figure 3). Genome sizes in the large body size isolines were not only larger on average than small body size populations, but they also exhibited a wider variation (Figure 3 and Figure 4). Therefore, large bodied individuals may actually have an increased genome size, yet others may only increase through increased cell-counts. The above suggests an idiosyncratic pattern for body size change. Body size change may, in fact, be a complicated interaction of cell-count and cell size, as suggested by *Drosophila* populations in Calboli, Gilchrist, Partridge and Fry [18]. 

It is likely that we see this pattern of increased variation in isolines and not the larger populations due to a reduction in selection efficacy attributed to lower effective population size and increased instances of genetic drift [33,45,46]. Increased selection efficacy in larger populations suggests that large changes in genomic structure can be detrimental and may provide a poor route to rapidly evolving a larger body size. Changes in genome size can dramatically influence the overall structure and architecture of the genome through insertion of new elements [47,48,49,50,51], introduction of heterochromatic regions [52], or even introduction of entire chromosomes [53,54]. Even small genome size changes can have significant effects on hybrid incompatibility. For example, insertion of inactive elements, such as pseudogenes, may be a factor leading to hybrid incompatibility between species [55]. These changes permitted by drift may result in genome instability within lines, as well as other impacts on the fitness of an organism. This thought is supported by the results of the *Drosophila* Genome Research Panel (DGRP), in which lines with larger genome sizes sometimes had larger variation in genome size than those with smaller genomes, suggesting higher instances of genome instability [4]. Additional work with these DGRP lines found significant relationships with genome size and fitness components when reared at different temperatures [5]. The lower variation in genome sizes we see here in small versus large body size selected isolines suggests that a small body size may constrain genome size. Small body size has been shown to reduce fitness by reducing fecundity [56,57,58]. Therefore, further constraint on fitness associated with genome size may not be tolerated. This constraint may be lifted in the case of large bodied populations, resulting in a potential, but not a guarantee, for change in genome size. This pattern of change in genome size where effective population size is low has been suggested previously [33]. Therefore, future studies should address this question of genome size change when selecting for body size in terms of controlled low and high effective population sizes.

While this work does not show a remarkable change in genome size as the result of selection, it does provide some limited insight to the effects of selection experiments and effective population sizes on genome size change. Effective population size may play an important role in the ability of genome size to change in these experiments. Higher instances of genetic drift from low effective population sizes in the *D. melanogaster* isolines may explain the only directional change in genome size we find. In this case, there was some evidence for weak directional selection, in which large-bodied flies increased in genome size, suggesting relaxation of the constraint on genome size change seen in the small-bodied flies. It is important to note, however, that size of an organism is more complicated than just investigating factors that may influence cell size or cell-count [59]. While there are known relationships between body size and development time, this relationship has been controlled in the *Drosophila* study. Small and large body size flies were cultured on the same day and generations were allowed to synchronize, controlling for this known contributor to body size. In the case of selection for development time, a reduction in genome size variation by generation was seen, with convergence on a mean genome size across treatments, suggesting stabilizing selection on genome size that is unconnected to development time. While a reduction in variation in a selection experiment is expected, lower variation by generation in development time was not documented in the City*Selection interaction. It is possible the scale of this study does not have enough resolution to show the changes happening at the genomic level. Genome size estimation, while accurate, is at the mega base pair level, only accounting for relatively large changes in size. It is possible, that change is occurring at a much slower and gradual rate, as observed between *Drosophila* species [60,61,62,63]. Large changes in size are more likely to occur with activation of transposable elements and other mechanisms, which are less predictable [49,64,65,66,67]. 

## Figures and Tables

**Table 1:** Variance component analysis results of genome size and development time in *C. macellaria* populations. As no differences were found among genome sizes, all strains were merged for the variance analysis of genome size. Variance decreases by generation for City*Selection in genome size. Contrastingly, variance in development time for City*Selection was maintained. Variance increases in selection by generation, verifying successful divergence in phenotypes.

Trait	Gen.	City	Selection	City*Selection	Error
Genome Size	1	0	0	272.1	467.6
	10	0	9.3	31.7	291.9
	32	8.6	5.7	17.4	83.5
Dev. Time	1	211.7	0	0	258.7
	10	0.4	684.8	114.7	169.7
	32	0	6929.8	152.8	482.3

**Table 2:** Average genome size estimates (Mbp) and development time with standard deviations (St. Dev) of *C. macellaria* under developmental selection. ‘N’ represents number of individuals measured. Development time information for Fast and Slow lines from each location at Generation 1 are the same as Generation 1 in Control.

	**College Station**						
		**Genome Size**			**Development Time**		
**Selection**	**Gen.**	**N**	**Mbp**	**St. Dev**	**N**	**Hours**	**St. Dev**
Control	1	11	531.26	18.33	1209	258.23	18.42
	10	8	524.29	18.9	1038	256.62	13.59
	32	10	539.72	7.03	1161	269.53	15.85
Fast	1	11	523.71	14.26	1209	-	-
	10	3	551.35	14.42	1100	241.88	8.26
	32	10	529.37	5.44	988	203.29	27.52
Slow	1	9	559.11	24.84	1209	-	-
	10	10	525.76	18.26	1125	310.33	10.79
	32	10	544.08	9.38	996	367.75	20.97
	**Longview**						
		**Genome Size**			**Development Time**		
**Selection**	**Gen.**	**N**	**Mbp**	**St. Dev**	**N**	**Hours**	**St. Dev**
Control	1	10	550.1	20.5	1003	284.61	14.61
	10	9	523.9	12.45	986	280.73	17.55
	32	9	531.91	3.03	1068	304.4	14.62
Fast	1	5	566.77	20.52	1003	-	-
	10	5	534.32	11.6	1015	264.85	15.28
	32	10	528.53	9.63	1044	193.08	32.67
Slow	1	6	538.92	27.41	1003	-	-
	10	9	541.21	19.31	974	299.86	13.06
	32	10	527.99	11.27	1151	366.33	13.57
	**San Marcos**						
		**Genome Size**			**Development Time**		
**Selection**	**Gen.**	**N**	**Mbp**	**St. Dev**	**N**	**Hours**	**St. Dev**
Control	1	9	543.15	18.37	1176	281.26	14.68
	10	3	538.83	7.49	990	281.04	18.13
	32	10	529.05	7.02	1032	280.39	24.54
Fast	1	10	550.58	23.28	1176	-	-
	10	7	540.5	16.64	1026	252.86	6.32
	32	10	531.49	8.03	847	194.67	20.86
Slow	1	12	528.45	26.06	1176	-	-
	10	10	533.21	20	1013	306.03	10.03
	32	10	539.87	15.29	958	365.95	21.35

## Supplementary Materials

The following are available online at https://www.mdpi.com/2073-4425/11/2/218/s1, Figure S1: Effect of selection for development time in different populations of the blow fly *Cochliomyia macellaria*, Figure S2: Genome size change after selection for development time, Figure S3: Genome size variation decreases with increasing number of generations of selection in *C. macellaria.*
