# Supplementary material for: Effect of Phenotype Selection on Genome Size Variation in Two Species of Diptera
Source: Genes (Basel). 2020 Feb 19;11(2):218. doi: 10.3390/genes11020218 (PMC7074110; doi:10.3390/genes11020218)
Supplement: Supplementary file 1 [file genes-11-00218-s001.pdf]

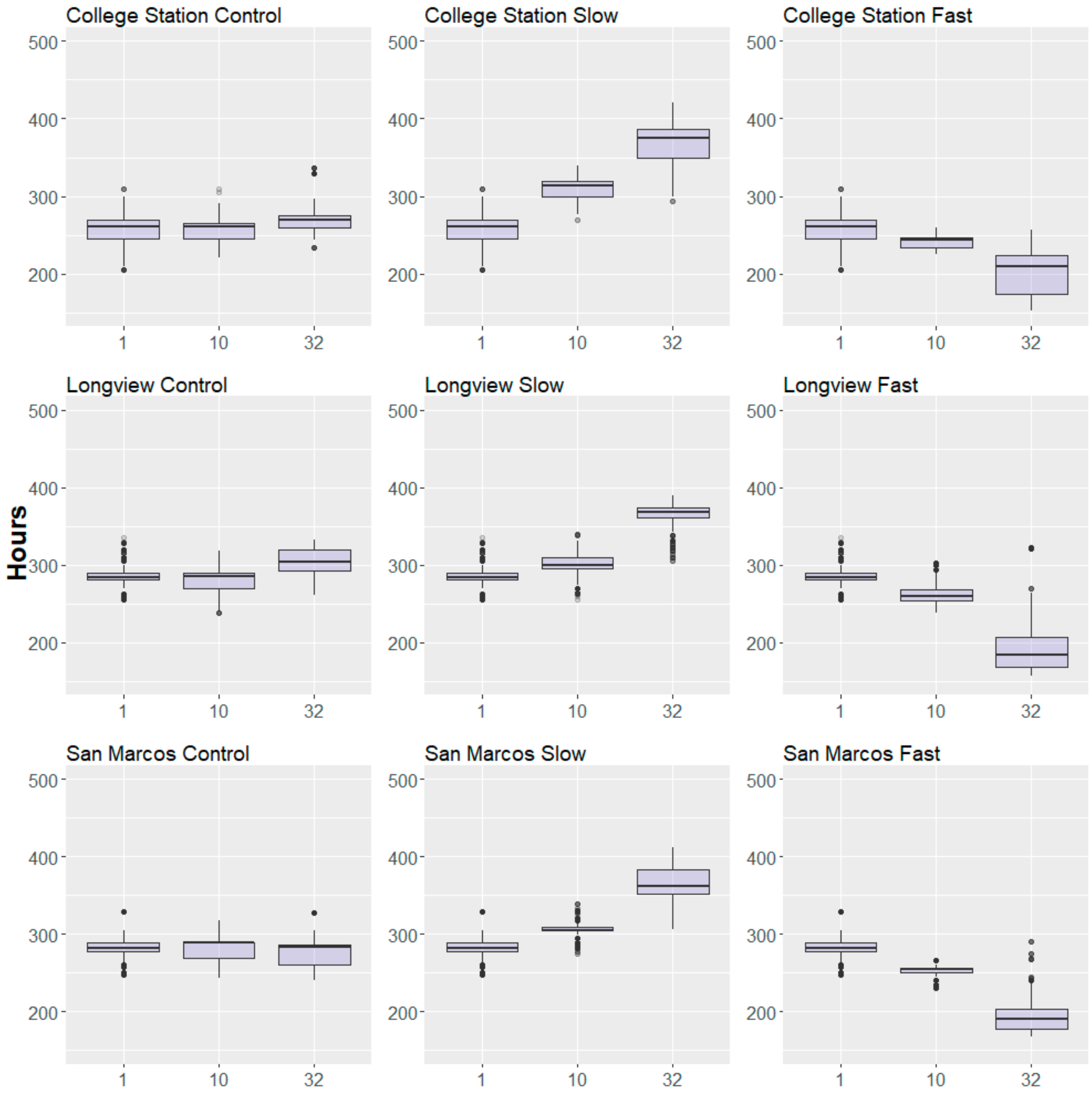

Figure S1: Effect of selection for development time in different populations of the blow fly *Cochliomyia macellaria*. Hours for development for each selection treatment and city at generations 1, 10, and 32 are plotted in box and whiskers format. No change in development time was seen in control lines, increases in development time was seen in slow selected lines, and decrease in development time was seen in fast selected lines.

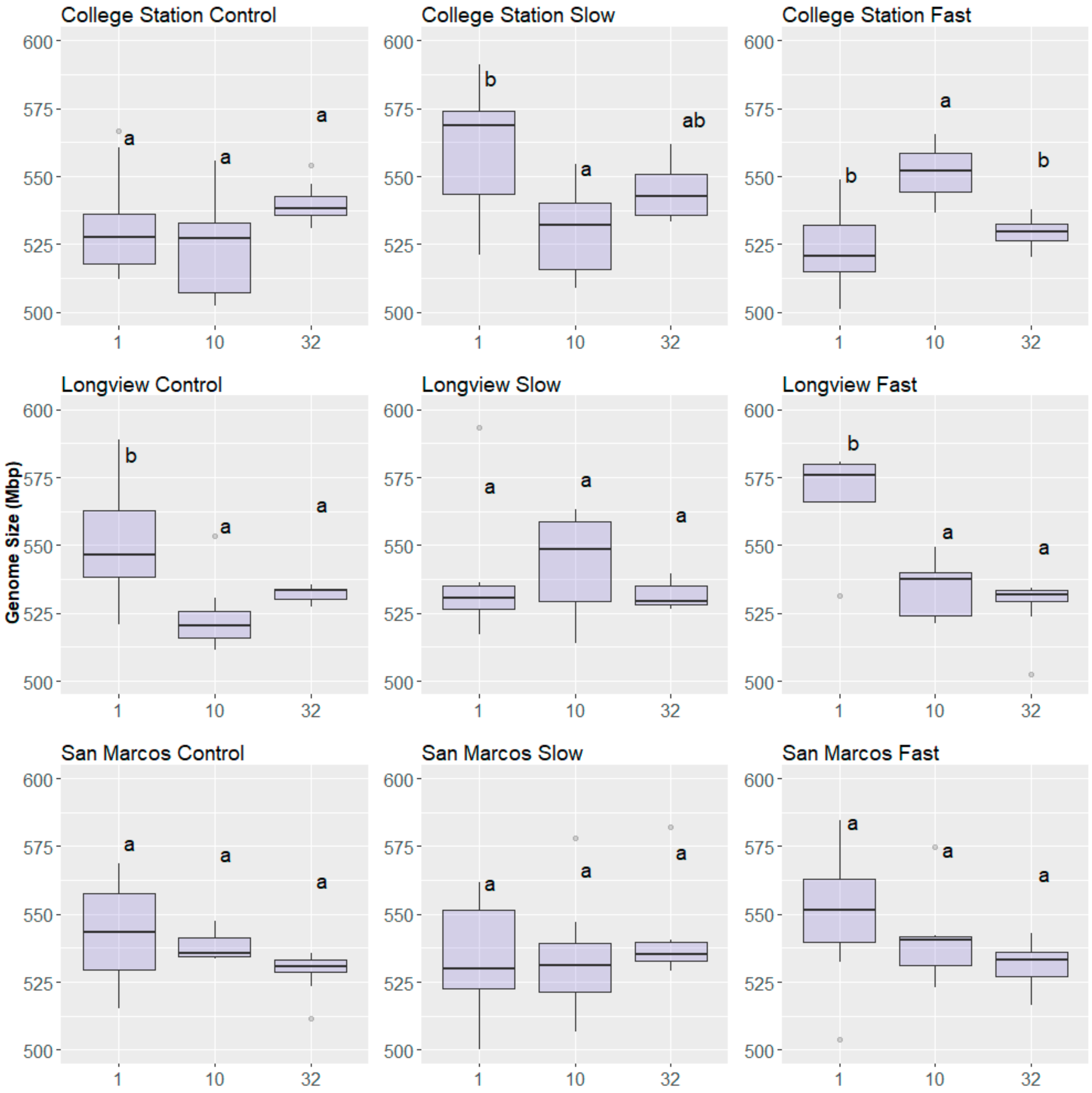

Figure S2: Genome size change after selection for development time. Genome size was plotted for each selection treatment for each city. Different letters above each box represent values significantly different according to Tukey HSD with the confidence level set at 0.95. There is a consistent reduction in genome size variation with increasing generations of selection. After 32 generations of selection, all groups converged on a similar genome size, approximately 530 Mbp.

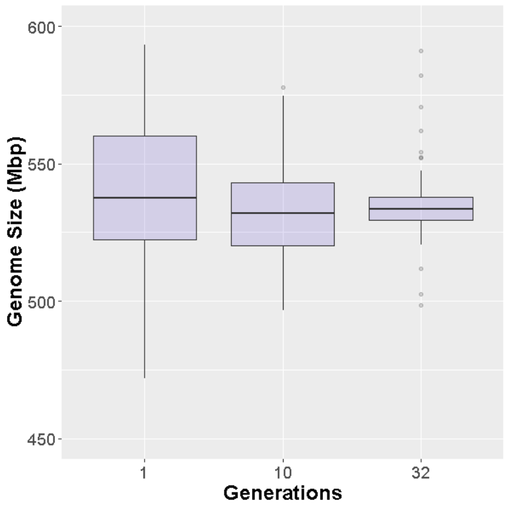

Figure S3: Genome size variation decreases with increasing number of generations of selection in *C. macellaria*. Genome size data was pooled between selection treatments and locations since there were no significant discernible patterns. This data was plotted in box and whisker plots to represent decreasing variation with generations of selection and the convergence on a mean genome size of approximately 530 Mbp.

Table S1: Genome size estimates for body size selected *Drosophila* lines

Selection	Line	Sex	N	GS (Mbp)	St Dev. GS	Rel. Cell Count	St. Dev. Cell Count
L1	F	F	4	184.13	4.51	1.21	0.18
		M	5	200.12	11.62	1.16	0.22
	MM	F	4	189.82	9.32	1.39	0.53
		M	4	185.70	5.98	0.98	0.05
	H	F	5	193.61	9.29	1.15	0.24
		M	4	188.54	7.20	1.02	0.10
	FF	F	5	199.52	17.61	1.05	0.18
		M	5	187.53	10.26	1.12	0.20
L2	F	F	5	185.22	5.13	1.25	0.29
		M	5	198.54	9.33	1.15	0.11
	TT	F	5	188.73	8.58	1.07	0.17
		M	3	184.03	0.41	1.04	0.10
	L	F	5	190.36	7.07	1.11	0.12
		M	3	186.03	2.17	1.06	0.06
	LL	F	5	201.99	11.74	1.05	0.18
		M	4	189.24	9.82	1.13	0.08
S1	G	F	5	182.44	2.61	0.86	0.53
		M	5	182.05	2.99	0.80	0.10
	Y	F	4	183.60	3.69	0.99	0.32
		M	5	187.01	4.54	0.81	0.24
	YY	F	4	185.52	1.92	0.95	0.11
		M	3	186.59	2.99	0.94	0.11
	AA	F	5	185.86	1.09	1.03	0.25
		M	4	183.54	2.20	0.79	0.10
	BB	F	5	185.86	2.00	0.88	0.30
		M	3	187.47	5.18	0.85	0.03
	W	F	4	187.17	7.34	0.91	0.15
		M	4	187.26	4.38	0.91	0.09
S2	FF	F	4	184.43	1.90	0.99	0.37
		M	4	185.93	6.25	0.82	0.21
	V	F	5	184.57	3.34	1.02	0.05
		M	5	186.00	3.00	1.02	0.13
	F	F	4	187.37	6.74	0.82	0.25
		M	4	183.39	1.19	0.78	0.12
Outbred C1	-	F	4	176.55	0.07	0.77	0.07
Outbred C2	-	F	4	176.83	0.05	0.73	0.05
Outbred L1	-	F	4	177.39	0.08	0.89	0.08
Outbred L2	-	F	4	177.04	0.03	1.01	0.03
Outbred S1	-	F	4	176.25	0.09	0.66	0.09
Outbred S2	-	F	3	177.47	0.04	0.53	0.04
